# Customer Cooperation and Employee Innovation Behavior: The Roles of Creative Role Identity and Innovation Climates

**DOI:** 10.3389/fpsyg.2021.639531

**Published:** 2021-06-03

**Authors:** Jian Zhou, Jian Yang, Xue Zhou

**Affiliations:** ^1^Department of Business Management, School of Business, Qingdao University, Qingdao, China; ^2^Department of Computer Science and Technology, Ocean University of China, Qingdao, China

**Keywords:** creative role identity, employee innovation behavior, high star hotel, innovation climate, customer cooperation

## Abstract

Employee innovation behaviors lay the foundation for sharing economies and are of importance to business success, especially for service sector firms such as hotels. This study examines the relationship between customer cooperation and employee innovation behavior (EIB) by focusing on the mediating role of creative role identity and the moderating role of innovation climate. Drawing on resource based theory and role identity theory, we propose that customer cooperation enhances creative role identities and EIB, and the relationship between creative role identities and EIB is stronger when innovation climates are described as “high” rather than “low.” A total of 213 respondents in high star hotel were selected for questionnaire survey in this study. The results indicate that Customer cooperation is positively related to EIB. Customer cooperation positively affects EIB partially through creative role identities and innovation climate strengthens the direct effect of creative role identities on EIB and the indirect effect of customer cooperation on EIB through creative role identities. Theoretical and practical implications were also discussed.

## Introduction

With the emergence of peer-to-peer platforms and sharing economies, traditional hotels have suffered (Zervas et al., 2017; Xie et al., 2019). Such hotels are now faced with intense competition from rival companies and challenged by the new economy. For service sector firms such as hotels, innovation has been key to improving competitiveness and performance (Campo et al., 2014). Due to the special nature of the service industry, innovation in hotels is often about service improvements that rely on employees (especially frontline employees in contact with customers) more than professionals (Ottenbacher, 2007; Li and Hsu, 2016b; Hassi, 2019). It has been widely accepted that employee innovation behavior is beneficial to both service sector firms and employees themselves (Martínez-Ros and Orfila-Sintes, 2012). It can provide new products and improve service processes (Enz and Siguaw, 2003; Orfila-Sintes and Mattsson, 2009; Su, 2011), and influence the competitive advantage and performance of hotels (Ottenbacher, 2007; Pivčević and Petrić, 2011). A significant number of studies have tried to find the influencing factors for these positive consequences (Chang et al., 2011; Tu and Lu, 2013; Li et al., 2020).

However, most of the research into employee innovation behavior has been conducted in manufacturing industries rather than in a service sector context (Li and Hsu, 2016a; Nazir et al., 2018). Innovation behavior in the service sector, by its nature, relies on service encounters—especially interactions between frontline employees and customers (Kelley et al., 1990; Ajitha et al., 2019). More recently, researchers have focused on open service innovation logic, which views customers as external resource providers during the innovation process (Mina et al., 2014; Wang et al., 2020). Following this logic, employee innovation behavior not only relies on an individuals’ information and skills, but also on outside resources and capital. This means employees need to seek help from outside of company structures—for example from customers to access resources and information useful to innovation (Foss et al., 2011; Schaarschmidt et al., 2018). Customer behaviors have been seen as important antecedents to employee innovative behavior. Categorized as a voluntary performance, customer cooperation can provide external support for employees (Bettencourt, 1997; Geilinger et al., 2020). It can provide information that helps improve service processes, facilitates the development of more attractive services, and reduces the costs of implementing new products and services (Sánchez-González and Herrera, 2014). From the perspective of social exchange, customers that cooperate and provide support and assistance to employees are rewarded with superior customer service by way of any resultant innovation (Chen, 2016). However, little research has been conducted into the impact of customer cooperation on employee innovative behavior within the service sector. This study attempts to fill this gap.

Drawing on role identity theory (Riley and Burke, 1995; Tierney and Farmer, 2011), the second goal of our research is to test the mediated role of creative role identity between customer cooperation and employee innovation behavior. According to role identity theory, employees evaluate themselves through a sense of role identity and they evaluate their abilities partially based on how they see themselves (Gist and Mitchell, 1992; Song et al., 2015). A creative role identity refers to employees who view themselves as imaginative people that can provide imaginative service products (Farmer et al., 2003; Wang and Cheng, 2010; Wang et al., 2014). When employees see themselves as embodying a creative role identity, in order to reduce the considerable social and personal costs (such as those aroused by misunderstanding), they often protect their self-image by providing more creative service solutions, which may lead to employee innovation behavior (Farmer et al., 2003; Wang and Cheng, 2010). Besides, according to role identity theory, a person’s sense of role identity is drawn from two main sources; one is their attitude toward social relationships and the other is the associated self-image (Riley and Burke, 1995; Farmer et al., 2003). To the extent that relevant inputs from others verify and support role identities, it is likely that customer cooperation is very important. Research has shown that role identity reflects an internalized set of role expectations (Farmer et al., 2003). Employees can, through service encounters (especially face-to-face service), perceive that cooperative customers accept them creatively delivering excellent services. This leads to employees defining themselves as creative people and promotes innovative behavior (Mahmood et al., 2019). We propose that customer cooperation can, at least in part, promote employee innovation behavior because customer cooperation supports creative role identities.

Further, we examine the moderating role of innovation climates on the relationship between creative role identities and employee innovation behavior. An innovation climate is described by how much an environment encourages risk-taking behavior, how it allocates resources, and how much it facilitates the pursuit of new ideas (Scott and Bruce, 1994; Jaiswal and Dhar, 2015; Danvila-del-Valle et al., 2018). Research has noted that the creative role identity tends to personalize contextual feedback, so the innovation climate may be a critical context for the creative process (Farmer et al., 2003). In addition, creative role identities reflect a set of role expectations. The innovation climate can be viewed as organizational expectations or the extent of innovation support. The innovation climate may make employees understand the value of creativity and organizational expectations–enhancing the effect of creative role identities and promoting high levels of creativity (Charbonnier-Voirin et al., 2010; Wang et al., 2013; Karatepe et al., 2020). We propose that, within high-level innovation climates, creative role identities are more likely to support employee innovation behavior.

In developing and testing this model, our research makes three major contributions to the literature. First, we examine customer cooperation as a predictor of employee innovation behavior within the hotel industry. It is likely to advance current understanding of innovation behavior within service encounter contexts as well as the external resources involved in open service innovation logic. Second, drawing on role identity theory (Tierney and Farmer, 2011), we examine the mediation mechanism (in the form of creative role identities) that operates between customer cooperation and employee innovation behavior–the “black box” underlying the effects of customer cooperation. Third, few studies have noted the organizational expectations surrounding role identities. This study addresses this by examining the moderating effects of innovation climate on the relationship between creative role identities and employee innovative behavior. This provides empirical evidence for the organizational sources of role identity theory.

## Theory and Hypothesis

### Employee Innovation Behavior

Employee innovation behavior is a cornerstone of organizational innovation, which is critical to the growth of hotel companies, their service quality, and customer satisfaction (Janssen et al., 2004; Campo et al., 2014; Li and Hsu, 2016b; Xu and Wang, 2020). Due to the characteristics of hotel service (intangibility, simultaneity of provision and consumption, customer involvement), employee innovation in hotels has different features to employee innovation in the manufacturing sector (Raub and Liao, 2012; Martín-Rios and Ciobanu, 2019). EIB in hotels relies more on employees than professionals (Ottenbacher, 2007; Ma et al., 2020). This means frontline employees can contribute innovative processes themselves rather than through a research department (Schaarschmidt, 2016). EIB in hotels also highlights the importance of customer-employee interactions, making customer service encounters of critical importance (Kaminakis et al., 2019). EIB in hotels is also often inspired or stimulated by customers and relies on the experience of individuals (Guisado-González et al., 2014; Lai et al., 2014). Customers provide both the resources and the expectations for innovation. In contrast to the manufacturing sector, EIB in hotels can attained without any special measures, which means it is an organizational source of new ideas and knowledge with which to serve the customer (Kim and Lee, 2013). From these four features it can be seen that, although EIB is an individual level concept, it can’t thrive without the support and resources of others especially from customers.

Employee innovation behavior is key to service sector firms’ performances and long-term survival, and it has provoked continuing research interest (Kim and Lee, 2013). Given the features of EIB, researchers have studied the precursors to EIB within the service sector. Individual-level factors are considered to be important. According to previous studies, employees with positive emotions (Staw et al., 1994; Lin and Lin, 2017), proactive personalities (Chen, 2011), profound knowledge (Chang et al., 2011), and an innovation cognitive style (for example, self-regulation and self-efficacy) (Beeftink et al., 2012) are more likely to perform innovatively. Second, EIB is dependent organizational-level factors. A firm’s resources, financial supports and reward (for example, innovation funds), structural and cultural contexts (innovation climates), and leadership style (as characterized by transformational leadership, empowering leadership, and servant leadership) are all important factors that influence EIB (Slåtten and Mehmetoglu, 2015). Third, due to work process related features, job characteristics have also been seen as foundational to EIB (Tu and Lu, 2013; Li and Hsu, 2016a). These characteristics include job complexity, job autonomy, skill variety and job demands, which directly, or through other factors, influence EIB (Oldham and Cummings, 1996; Coelho and Augusto, 2010; Robertson and O’Reilly, 2020).

### Customer Cooperation and Employee Innovation Behavior

The concept of customer cooperation has been viewed as an important part of customer’s experience, customer satisfaction, and the perception of quality (Bettencourt, 1997; Sánchez-González and Herrera, 2014). As a typical service industry, customers and employees in hotels interact more than in other industries and the working conditions of servers are unnecessarily more precarious than those in other service industries (Ariza-Montes et al., 2019). This has implications for others (Bettencourt, 1997; Suan and Nasurdin, 2013). In the hotel industry, customers want to enjoy a more comfortable service and employees want to provide excellent service. The hotels want to gain innovative capacity (Hernández-Perlines et al., 2019). Solutions for these demands can be delivered through employee innovative behaviors, which can also enhance the competitiveness of hotels (Ottenbacher and Harrington, 2007; Cismaru and Iunius, 2019). Customers can be seen by service sector employees as a vital source of work related activities, because the employees’ main task is to meet the needs of customers and leave them satisfied (Korczynski, 2003). EIB can therefore be considered as a value creation process. It can provide new products and improve service processes (Su, 2011). However, employees need the motivation and external resources necessary to support this process.

The attitude-intention-behavior framework shows that customer cooperation can affect employees’ service goals, work experience, and job attitude (Limpanitgul et al., 2013). Customer cooperation means that clients add extra effort to the service process and this needs to be rewarded by the hotels or employees, raising customer expectations. Employees’ service goals are therefore affected by providing better service that meets the expectations of cooperating customers (Chen, 2011). Second, customer cooperation can affect an employee’s subsequent work-related experience by helping employees become more familiar with the service scene. In turn, employees are able to spend more time on innovation than on daily work (Limpanitgul et al., 2013). Third, employees’ job attitudes can be affected by peers such as co-workers (Chiaburu and Harrison, 2008). Customers can be seen as a special type of co-worker in hotels. The positive behavior of customers can bring satisfaction and organizational commitment, while the negative behavior of customers can cause both poor work and antagonism (Harrison et al., 2006; Harter et al., 2020). With customer cooperation, service sector employees set higher service goals, receive a wealth of work experience, and develop a more positive job attitude. These attitudes and intentions encourage employees to seek better ways to serve their customers, promoting EIB.

Value co-creation, defined as co-creation of personalized experiences with customers, implies customers play important roles in this process. According to the definition, EIB is one kind of value creation, created by the employees and flowing toward the customer (Agrawal and Rahman, 2015). If customers are clear about their service expectations and employees can meet those expectations, then the co-creation of value is manifest. According to previous research, customers can play various roles within EIB, such as co–designer, co-ideator, co-innovator, co-evaluator, and experience creator (Bitner et al., 1997; Najafi-Tavani et al., 2020). Co-designer and co-ideator hotel customers make services more personalized and convenient. The wide experience and specific knowledge of customers can combine with existing employee experience to promote the innovation behavior. In service dominant logic, customers can also be co-evaluators and experience creators. The employees service quality is evaluated by customers and either value-in-exchange or value-in-use can benefit from the latent perceptions and preferences of customers and capitalize on them (Vargo and Lusch, 2004). Deeper understanding requires intense communication with co-innovator customers but can enhance the effectiveness of feedback systems in hotels. In these terms, the customer can be described as a “prosumer” (Xie et al., 2008).

Customer cooperation also provides motivation and resources for employees to act on innovation behaviors. Previous studies have shown that perceived insider status and organization-based self-esteem can affect EIB (Lee and Hyun, 2016). When customers display cooperation behavior by, for example, following guidelines, employees feel respected and seek ways to improve service (Sui and Wang, 2014; Ng, 2016). Also, customers can contribute properties such as physical resources, financial resources, human cultural resources, informational resources, and social resources to employees seeking to improve services. Agrawal and Rahman (2015) identified three kinds of physical resource; energy, emotion, and strength. These resources provide emotional support for EIB. Further, customer cooperation provides financial and informational supports such as physical space, information sharing, and knowledge transfer, which can lead to more innovative behaviors (Foss et al., 2011). Customers also provide relational and social resources such as customer communities and commercial relationships (Vafeas and Hughes, 2020). Therefore, the following hypothesis is proposed.

*H1*: *Customer cooperation is positively related to employee innovative behavior.*

### The Mediating Role of Creative Role Identities

Identity theory suggests that individuals see themselves in terms of a role identity. This determines “who they are” and “what they should do” to meet the requirements of the role. Creative role identity was first described using role identity theories by Farmer et al. (2003) as employees who view themselves as creative people who can provide creative service products. According to previous research, a person’s role identity has two main sources: feedback about the self from social relationships, such as from co-workers, and self-identification and self-regulation (Riley and Burke, 1995; Grube and Piliavin, 2000; He et al., 2020).

In this research, we argue that creative role identities mediate the relationship between customer cooperation and employee innovative behavior. According to role identity theory, the feedback and expectations of others are important sources for individuals self-identification (Farmer et al., 2003). For hotel employees, expectations of creativity come from two main groups; leaders and customers. Creativity here means ideas leading to improvements of service levels. Customers like to be provided with better service than expected, which means conventional service styles and service processes can’t meet their needs. To meet these needs, hotel employees make themselves more creative. Recent studies have shown that co-workers influence creativity through encouragement, support, open communication, and informational feedback (Zhou and George, 2001; Malingumu et al., 2016). Customer cooperation entails the provision of service information, communication with frontline employees, and support and encouragement of creativity and better service. The sense of creative role identity is formed through this cooperation (Jessen et al., 2020). In addition, the associated self-view and self-regulation are also important sources for creative role identity. With customer cooperation, hotel employees can experience positive behavior from customers and, in response, view and define themselves as more creative (Jessen et al., 2020).

As noted by Gist and Mitchell (1992), employees make determinations partially based on how they see themselves. A creative role identity means the individuals view themselves as a creative person who should do creative jobs at work. Hence, with a highly creative role identity, employees pay more attention to creative service information to provide valuable resources for EIB. Wang et al. (2014) found that employee creative role identity is positive related to employee creative self-efficacy. Identity and efficacy are both complicated self-perceptions, which allow employees to confidently remove inherent obstacles to creative activities and implement more creative tasks. The major purpose of hotel employees is to provide excellent service and satisfy customers (Hu et al., 2009). The creative role identity can make creativity and service awareness become more intrinsic to employees, make them more sensitive to contextual supports for their service quality, and encourage higher levels of creative work involvement (Kim and Shim, 2018).

According to Erkutlu and Chafra (2015), employees with strong role identities are extremely sensitive to external support. They tend to treat external stakeholders as important supports to their innovation implementation behavior. In Wang et al. (2014) paper, a creative role identity was found to act as a bond between transformational leadership and employee creativity. They noted that it can inspire employees to adopt creative approaches that exhibit high levels of self-reinforcement instead of being dependent on external reward. In this research, we propose that enhanced creative role identities resulting from customer cooperation may improve the relationship between customers and employee behavior within the hospitality industry. When employees think of creativity as a central component of their job, they are more likely to act creatively and proactively to meet customer demands in a different way. However, those employees lacking the impetus provided by a creative role identity might lose sensitivity to innovation resources and innovative service performance will be limited (Farmer et al., 2003). As scholars have argued, a creative role identity can affect creative self-efficacy, which can lead to even greater creativity (Gushue et al., 2006; Schmitt et al., 2018). When confident employees have creative self-efficacy, they may able to complete service work more efficiently and creatively (Newman et al., 2018). For these reasons, we argue that customer cooperation promotes creative role identity, which provides the motivation and resources for EIB. Thus, we propose the following hypothesis.

*H2*: *Creative role identities mediate the relationship between customer cooperation and employee innovative behavior.*

### The Moderating Role of Innovation Climates

Innovation climates are an indicator of how employees set their role expectations. Such environments encourage risk-taking behavior, the allocation of resources, and the pursuit of new ideas (Scott and Bruce, 1994; Jaiswal and Dhar, 2015; Popa et al., 2017). This results in higher level of motivation, commitment, and employee engagement (Shanker et al., 2017; Zhang et al., 2018). As noted by some researchers, a creative role identity not only tends to personalize contextual feedback, but also depends upon a combination of both personal qualities and work environment factors. From the work environment perspective, we hypothesize that innovation climates can affect the effectiveness of creative role identities. A high-level innovation climate may make employees perceive the value of creativity and organizational expectations and enhance the effect of creative role identities on levels of creativity (Charbonnier-Voirin et al., 2010; Wang et al., 2013). Because the core of a creative role identity is a transformation of the consciousness of external expectations and role requirements into a self-awareness through an identity regulation mechanism, such employees try to keep personal attitudes and behavior aligned with the social roles they play in social interaction. As noted by Brenner et al. (2014), when employees assume a clear role position, they feel pressure from others and the environment that ensures they act within their role. In other words, innovation climates also strengthen the creative and innovation role identities of hotel employees.

Within a high-level innovation climate, the creative role identity of hotel employees may play a more significant role in promoting EIB. On the one hand, employees will feel a driving force from their organization and their creative role identity is strengthened (Shanker et al., 2017). On the other hand, hotel employees feel pressure from the innovation environment, which forces them to overcome difficulties and solve problems according to the role identity. High-level innovation climates encourage employees to produce good ideas and views. These ideas and views can be transformed into employee innovative behavior to some extent. In low-level innovation climates, creative employees lose the supervision and support of their organization. In such a situation, new ideas and new behaviors are difficult to produce and the effectiveness of employee innovation behavior is restrained. Consequently, we hypothesize the following.

*H3*: *Innovation climates moderate the relationship between creative role identities and employee innovative behavior such that the positive relationship is stronger when innovation climates are high-level rather than low-level.*

The above arguments represent an integrated framework in which creative role identities mediate the impact of customer cooperation on employee innovative behavior, and innovation climates moderate the relationship between creative role identities and employee innovative behavior. Thus, it is logical to propose that the positive but indirect effect of customer cooperation on employee innovative behavior through creative role identities will be stronger among employees in high-level innovation climates. Hence, we conclude this section by proposing the following.

*H4*: *Innovation climates moderate the indirect effect of customer cooperation on employee innovative behavior through creative role identities, such that the indirect effect is stronger when innovation climates are high-level rather than low-level.*

**FIGURE 1:**
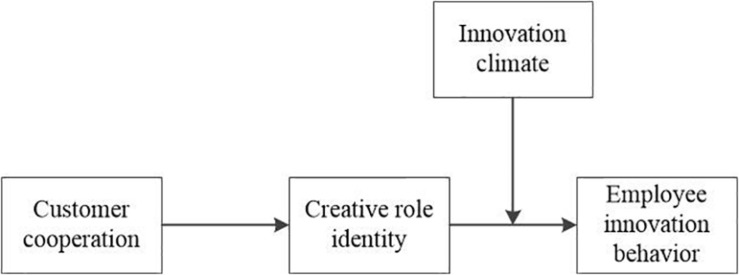
Depicts the conceptual model in this study.

Figure 1 depicts the conceptual model in this study.

## Materials and Methods

### Sample and Procedures

The main survey was carried out in Hangzhou, China, from September to October in 2017. As evidenced the city of Hangzhou having just held the G20 summit, and its reputation as China’s most beautiful and dynamic city, the hotels in Hangzhou are regarded as giving good service and with having innovative employees. Therefore, surveying hotel employees in Hangzhou may provide implications for other areas of China. The researchers contacted the CEO of ZTG, one of the largest hotel groups in China with more than 20 four and five-star hotels in Hangzhou. Besides, the author has maintained a long-term good relationship with the head and some managers of ZTG Group, the accuracy and authenticity of the collected data can be better ensured. Managers from these hotels agreed to participate in the survey and helped to collect data. Questionnaires were distributed directly to the employees by hand or by using the human resources departments serving the hotels. Before the survey process the researchers briefly introduced the purpose of this study and promised the questionnaires would only be used for academic research. During the survey process, the researchers had no contact with the respondents and were careful to leave enough time for employees to complete the questionnaires.

The researchers distributed 300 questionnaires. After eliminating questionnaires where over five questions were unanswered, a total of 213 valid questionnaires were left. The valid response rate was 72.3%. Among the 213 respondents, 41.8% were males and 55.4%were females. 9.9% of were 25 or younger, 26.8% were between 25 and 35, 36.2% were between 36 and 45, and 26.3% were 46 or older. Most respondents held a high school degree or below (62.4%), 29.1% held a junior college degree, and 7.5% had a bachelor’s degree or higher. The respondents were first-line employees (68.5%), supervisors (25.8%), and department managers (4.7%). All of the hotels were four- and five-star hotels and most of them had operated for 6–10 years (69%). Table 1 demonstrates the characteristics of the respondents.

**TABLE 1:** Characteristics of the respondents (*n* = 213).

	**Value**	**Number of responses**	**Percentage (%)**
Gender	Male	89	41.8
	Female	118	55.4
	Total	207	
Age	25 or younger	21	9.9
	25–35	57	26.8
	36–45	77	36.2
	46–55	55	25.8
	55 or older	1	0.5
	Total	211	
Education	High school or below	133	62.4
	Vocational school	62	29.1
	University	15	7.0
	Master’s/Ph.D.	1	0.5
	Total		
Seniority	First-line employee	146	68.5
	Supervisor	55	25.8
	Department manager	10	4.7
	Total	211	
Hotel age	Under 1	12	5.6
	1–2	13	6.1
	3–5	33	15.5
	6–10	147	69.0
	Above 10	6	2.8
	Total	211	

### Measures

The survey was administrated in Chinese. The measures used in this study were originally designed in English and were then translated into Chinese with the assistance of two Ph.D. candidates. To ensure equivalence of meaning (Brislin, 1980), these measures were back translated into English by another Ph.D. candidate. Two management professors then checked the instrument and made modifications to correct for discrepancies. To ensure content validity, we consulted three employees in the hotels to ask for their advice and modified according to their comments.

#### Customer Cooperation

We adopted a five-item customer cooperation scale developed by Limpanitgul et al. (2013) to measure the extent that customers like to cooperate. Response options ranged from 1, “strongly disagree” to 5, “strongly agree.” Items included: “Customers try to keep the hotel clean (e.g., not leaving rubbish in the floor),” “Customers carefully observe the rules and policies of the hotel,” “Customers always treat the hotel‘s staff with kindness and respect,” “Customers do things to make my job easier (e.g., they are ready to accept substitutes when something is not available)” and “Customers endeavor to avoid requesting tasks that are not required of me.” Cronbach’s alpha for this measure was 0.84, indicating adequate reliability.

#### Creative Role Identity

We adopted a three-item Creative Role Identity Scale (Farmer et al., 2003) to measure the extent to which the role of creative employee had been incorporated into self-identity. This scale has been validated in the Chinese context (Wang et al., 2014). Response options ranged from 1, “strongly disagree” to 5, “strongly agree.” Items included: “I often think about being creative,” “I have clear concept of myself as a creative employee,” and “To be a creative employee is an important part of my identity.” Cronbach’s alpha for this measure was 0.82, indicating adequate reliability.

#### Innovation Climate

A ten-item scale originally developed by Scott and Bruce (1994) and later modified by Jaiswal and Dhar (2015) was used to measure innovation climate. In order to describe clearly, we modified the reversed items. Response options ranged from 1, “strongly disagree” to 5, “strongly agree.” Sample items included: “Creativity is encouraged here,” “Our ability to function creatively is respected by the supervisor,” and “Around here, people are allowed to try to solve the same problems in different ways.” Cronbach’s alpha for this measure was 0.90, indicating adequate reliability.

#### Employee Innovation Behavior

A five-item scale originally developed by Matear et al. (2004) and Scott and Bruce (1994) and later modified by Hu et al. (2009) was used to measure EIB. Response options ranged from 1, “strongly disagree” to 5, “strongly agree.” Items included: “At work, I come up with innovative and creative notions,” “At work, I try to propose my own creative ideas and convince others,” “At work, I seek new service techniques, methods, or techniques,” “At work, I provide a suitable plan for developing new ideas,” and “At work, I try to secure the funding and resources needed to implement innovations.” Cronbach’s alpha for this measure was 0.88, indicating adequate reliability.

#### Control Variables

Following recent studies of creativity, we controlled for employees’ gender, age, education, and seniority to rule out their effects on task expertise and knowledge, which, in turn, could play role in determining EIB (Richter et al., 2012). We also controlled for hotel age to rule out effects on innovation climate.

### Common Method Bias

In order to reduce any problem of common method bias that may exist in this paper, during data collection the research group collected the data separately from the explained variables as far as possible. This paper also uses the Harman single factor test to test the homologous variance of the main variables involved. The results show that the first factor can account for 26.68% of the variance, and the cumulative can explain 58.80% of the variance. The variance explained by the first factor is less than half of the total variance. Therefore, there is no serious common method bias problem in this paper.

## Results

### Confirmatory Factor Analysis

We conducted a series of confirmatory factor analyses to evaluate the distinctness of the key variables. Following the suggestion of Hau and Marsh (2004), we first examined the baseline model (the four-factor model) that included four key variables: customer cooperation, creative role identity, innovation climate, and EIB. The four-factor model indices showed that the data fit well [χ^0^ ^2^(224) = 536.76, RMSEA = 0.07, GFI = 0.83, CFI = 0.91, TLI = 0.90, and SRMR = 0.07] and all the factor loadings were significant. To confirm the measurement model, the baseline model was contrasted with alternative CFA models. The alternative CFA models are shown in Table 2 and it can be seen that the four-factor model fitted the data considerably better than any of the alternative CFA models (Cheung and Rensvold, 2002). Hence, the discriminant validity of the four variables was confirmed.

**TABLE 2:** Results of confirmatory factor analysis for the measures of the variables studied.

	**χ^2^**	***Df***	**RMSEA**	**GFI**	**CFI**	**TLI**	**SRMR**
Null model ^a^	900.00	231	0.11	0.75	0.79	0.76	0.27
Four-factor model	536.76	224	0.07	0.83	0.91	0.90	0.07
Three-factor model ^b^	616.20	227	0.08	0.81	0.88	0.87	0.07
Three-factor model ^c^	620.17	227	0.08	0.81	0.88	0.86	0.07
Three-factor model ^d^	547.80	227	0.08	0.83	0.90	0.89	0.07
Two-factor model ^e^	629.60	230	0.08	0.81	0.88	0.86	0.07
One-factor model ^f^	1,007.40	231	0.12	0.65	0.76	0.72	0.09

*n = 213.*

*^*a*^In the null model, there is no relation between all measurements.*

*^*b*^Customer cooperation and creative role identity merged as a potential factor.*

*^*c*^Customer cooperation and employee innovation behavior merged as a potential factor.*

*^*d*^Creative role identity and employee innovation behavior merged as a potential factor.*

*^*e*^Customer cooperation, creative role identity and employee innovation behavior merged as a potential factor.*

*^*f*^All measurements merged as a potential factor.*

### Descriptive Statistics

Table 3 presents the means, standard deviations, and correlations of all the variables. As shown in Table 3, customer cooperation was positively correlated with creative role identity (*r* = 0.42, *p* < 0.01) and employee innovation behavior (*r* = 0.48, *p* < 0.01), employee innovation behavior was positively correlated with employee innovation behavior (*r* = 0.67, *p* < 0.01). Moreover, all the square root of average variance extracted (AVE) of the constructs are higher than the correlation coefficients, suggesting the discriminant validity is confirmed (Fornell and Larcker, 1981).

**TABLE 3:** Means, standard deviations, and correlations.

**Variables**	**1**	**2**	**3**	**4**	**5**	**6**	**7**	**8**	**9**
1. Employee gender	N/A								
2. Employee age	−0.04	N/A							
3. Employee education	−0.13	−0.23**	N/A						
4. Employee seniority	−0.11	0.06	−0.54**	N/A					
5. Hotel age	0.07	0.01	−0.07	0.24**	N/A				
6. Customer innovation	−0.18**	−0.01	0.04	-0.06	−0.06	(0.72)			
7. Creative role identity	−0.21**	−0.07	0.23**	0.23**	0.02	0.42**	(0.77)		
8. Innovation climate	−0.22**	−0.06	0.16*	013	−0.04	0.59**	0.60**	(0.76)	
9. Employee innovation behavior	−0.34**	−0.04	0.22**	0.19**	0.00	0.48**	0.67**	0.65**	(0.76)
MEAN	1.57	2.80	1.36	1.45	3.58	3.56	3.87	3.58	3.74
S.D.	0.50	0.96	0.57	0.65	0.88	0.75	0.76	0.68	0.74

n = 213; *Significant at the *p* < 0.05 (**p < 0.01) level.

*Bracketed values on the diagonal are the square root of average variance extracted value of each construct.*

*N/A indicates not suitable for analysis.*

### Model Design

Combined with theoretical deduction and research hypothesis, the regression model is established as follows:

(1) $$\text{EIB}=\alpha_{1}+\alpha_{2}\,\text{CC}+\alpha_{\text{i}}\,{\text{Control}}_{\mathrm{gender},\mathrm{age},\mathrm{edu},\mathrm{sen},\mathrm{Hage}}+\varepsilon$$

(2) $$\text{EIB}=\alpha_{1}^{\prime }+\alpha_{2}^{\prime }\,\text{CC}+\alpha_{3}\,\text{CRI}+\alpha_{\text{i}}^{\prime }\,{\text{Control}}_{\mathrm{gender},\mathrm{age},\mathrm{edu},\mathrm{sen},\mathrm{Hage}}+\varepsilon$$

(3) $$\text{EIB}=\alpha_{1}^{\prime \prime }+\alpha_{2}^{\prime \prime }\,C\,C+\alpha_{3}^{\prime \prime }\,C\,R\,I+\alpha_{4}^{\prime \prime }\,I\,C+\alpha_{7}\,C\,R\,I\times I\,C+\alpha_{i}^{\prime \prime }\,C\,o\,n\,t\,r\,o\,l_{g\,e\,n\,d\,e\,r,a\,g\,e,e\,d\,u,s\,e\,n,H\,a\,g\,e}+\varepsilon$$

### Hypothesis Testing

Hierarchical multiple regression analyses were conducted to test the first three hypotheses. To test Hypothesis 1, we conducted Model 3 (only five control variables) and Model 4 (the control variables and customer cooperation were entered in separate steps). As shown in Table 4, customer cooperation was positively related to employee innovation behavior (β = 0.44, *p* < 0.01). Thus, Hypothesis 1 was supported.

**TABLE 4:** Results of hypotheses testing.

	**Creative role identity**		**Employee innovation behavior**					
	**M_1_**	**M_2_**	**M_3_**	**M_4_**	**M_5_**	**M_6_**	**M_7_**	**M_8_**
**Control variables**								
Employee gender	−0.18	−0.11	−0.31**	−0.23**	−0.18**	−0.16**	−0.16**	−0.14**
Employee age	−0.06	−0.06	−0.02	−0.03	0.02	0.12	0.02	0.02
Employee education	0.10	0.07	0.12	0.09	0.05	0.04	0.04	0.03
Employee seniority	0.16	0.20**	0.09	0.14	−0.03	0.01	−0.02	−0.01
Hotel age	−0.00	0.00	−0.01	0.00	−0.01	0.00	0.01	0.01
**Independent variable**								
Customer cooperation		0.40**		0.44**		0.18**	0.08	0.09
**Mediation**								
Creative role identity					0.73**	0.66**	0.57**	0.59**
**Moderators**								
Innovation climate							0.23**	0.23**
**Interaction**								
CRI × IC								0.08*
R^2^	0.10	0.25	0.15	0.34	0.43	0.66	0.69	0.70
ΔR^2^	0.10	0.15	0.15	0.19	0.28	0.32	0.54	0.01
F	4.51**	11.34**	7.06**	16.80**	57.35**	54.57**	54.24**	49.42**
ΔF	4.51**	41.02**	7.06**	55.87**	262.84**	187.32**	113.18**	4.10*

n = 213; *Significant at the *p* < 0.05 (**p < 0.01) level.

*CRI = Creative role identity; IC = Innovation climate.*

To test Hypothesis 2, we conducted Model 2, Model 4, Model 5, and Model 6 (Baron and Kenny, 1986). Model 2 indicated that customer cooperation was positively related to creative role identity (β = 0.40, *p* < 0.01); Model 4 indicated that customer cooperation was positively related to employee innovation behavior (β = 0.44, *p* < 0.01); Model 5 indicted that creative role identity was positively related to employee innovation behavior (β = 0.73, *p* < 0.01); Model 6 indicted that, when both customer cooperation and creative role identity entered the model, the Beta coefficient between customer cooperation and employee innovation behavior became lower than Model 4 (β = 0.18, *p* < 0.01), while creative role identity was still positively related to employee innovation behavior (β = 0.66, *p* < 0.01). In addition, we tested the significance of the indirect effect using the bootstrap method developed by Williams and MacKinnon (2008). The results indicated that the indirect effect of customer cooperation on employee innovation behavior through creative role identity was significant (Estimate = 0.25, *p* < 0.01). Specifically, the 97.5% confidence interval of the indirect effect was (0.15, 0.40), not containing zero. Thus, Hypothesis 2 was supported.

**FIGURE 2:**
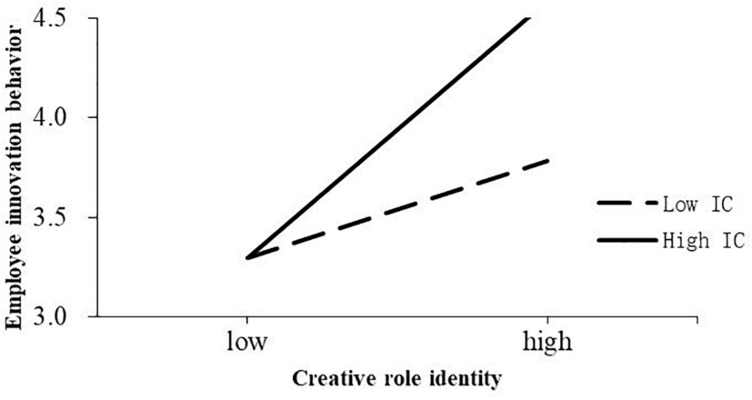
Interactive effects of creative role identity and innovation climate on EIB.

Hypothesis 3 proposed that innovation climates moderate the relationship between creative role identities and employee innovative behavior. To test Hypothesis 3, an interaction term was included in Model 8. As shown in Table 4, the interaction between CRI and IC was positively related to EIB (β = 0.08, *p* < 0.05), providing support for Hypothesis 3. A simple slope analysis was performed to show the interaction effect (Preacher et al., 2007). As shown in Figure 2, the interaction effect was obtained by plotting the estimates plus and minus one standard deviation of the means of moderating variables. The results showed that, when innovation climates were low-level, creative role identity had less impact on employee innovation and when innovation climates were high-level, the relationship was strengthened. Thus, Hypothesis 3 was supported.

The conditional indirect effect (Preacher et al., 2007) was examined to test Hypothesis 4 (moderated-mediation). The indirect effect of customer cooperation on employee innovation behavior through creative role identity varied significantly across different levels of innovation climate (conditional indirect effect = 1.03, *p* < 0.01). Specifically, when innovation climate was of a high-level (+1 standard deviation), the indirect effect of customer cooperation on employee innovation behavior through creative role identity was positively significant (conditional indirect effect = 0.83, *p* < 0.01); when innovation climate was of a low-level (−1 standard deviation), the indirect effect of customer cooperation on employee innovation behavior through creative role identity was negative (conditional indirect effect = −0.20, *p* < 0.05). The difference between the two conditions was 0.05 with 95% CI of (0.545, 2.120). Thus, Hypothesis 4 was supported, indicating that, when innovation climates are of a higher level, customer cooperation has a stronger relation to employee innovation behavior through creative role identity.

## Discussion

This study attempts to link customer cooperation and employee innovation behavior in the context of the hotel industry, bringing an interdisciplinary contribution to service and innovation research. A total of 213 respondents in high star hotel were selected for questionnaire survey in this study. The results indicate that customer cooperation positively influences employee innovation behavior. Also, we find that creative role identities mediate the direct effect of customer cooperation on employee innovation behavior. In addition, high-level innovation climates strengthen the direct effect of creative role identity on EIB and the indirect effect of customer cooperation on EIB. This model helps to explain how customer cooperation influences EIB and identifies the type of climate which is most conducive to innovation. These findings could provide some theoretic and practical implications.

### Theoretical Implications

This study contributes to the literature in three ways. First, we advance the literature surrounding customer cooperation by examining its outcomes in the hospitality industry. Prior studies show awareness that employee relationships with clients are important and an important source of knowledge to the innovation process (Kozioł et al., 2015), but little effort has been made to investigate the impacts on personal innovative behavior. Our research addresses this important gap by linking customers to employees and by exploring how customer cooperation promotes employee innovation behavior in hotels. We found that customer cooperation not only affects the motivation of employees (service goals, work experience and job attitude) (Limpanitgul et al., 2013), but that it also provides external resources that support employee innovative behavior. According to social exchange theory, exchanges with others may be potential innovation facilitators for employees because of the social networking opportunities (Li and Hsu, 2016a). Customer cooperation means exchange relationships or exchange of resources with hotel employees. For example, when customers show cooperative behavior, employees feel goodwill toward customers which, in turn, prompts employees to find better ways to serve customers. Further, as Agrawal and Rahman (2015) identified, customers can contribute physical resources, financial resources, human cultural resources, informational resources, and relational and social resources to aid service processes in hotels. This may lead to the new methods and new technologies that constitute employee innovation behavior.

Second, this study advances the literature surrounding customer cooperation by examining the mediating mechanism underlying the relationship between customer cooperation and employee innovation behavior. Prior studies show the relationship between customer satisfaction and employee behavior, however, its mechanism has not been explored in depth (Bambauer-Sachse and Helbling, 2021). Drawing on role identity theory, we adopt a new theoretical framework to explain the mediating role that creative role identity plays between customer cooperation and employee innovation behavior, and indicate that the self-role identity could be an important path linking customer cooperation and employee innovation behavior. These findings provide evidence for the view that the feedback and expectations of social others are important sources for individual innovation behavior. Thus, our study helps to reveal internal motivations and external resources of employee innovation in the hotel context and opens the “black box” underpinning the relationship between customer cooperation and employee innovation behavior. We also add to the literature surrounding customer cooperation and employee innovation behavior by including a role identity perspective. By exploring the mediating mechanism of creative role identities, our study finds that, once organizational expectations and role requirements translate into employees’ role positioning, employees can urge themselves to devote more time and energy to creating through intrinsic value recognition and integration mechanisms.

Third, we enrich the literature surrounding role identity by introducing environmental factors to explore the theoretical boundaries. In this study, we include innovation climate as a moderator in the process of creative role identity. Literature surrounding role identity has previously focused on the personalized and contextual (Farmer et al., 2003; Yuan and Woodman, 2021) and it represents an intrinsic motivation for behaviors. This study extends these findings by taking organizational climate into consideration, and by combining both personal qualities and work environment factors. We find that with high-level innovation climates, the positive relationship between creative role identity and employee innovation behavior is stronger. Further, the indirect effect of customer cooperation on employee innovative behavior through creative role identity is also stronger than when the innovation climate is of a low-level. By establishing the moderating influence of the organizational innovation climate, we extend prior research and identify a new and important boundary condition under which creative role identities affect employee innovation behavior.

### Managerial Implications

Our research has important practical implications for the hotel industry. Faced with intense competition and an emergent new economy, especially in the context of COVID-19, innovation has been key to improving competitiveness and performance for service sector firms like hotels (Campo et al., 2014; Zervas et al., 2017). Our study provides some suggestions for hotels seeking to improve employees’ innovation behavior to gain a competitive advantage. First, hotels should emphasize the value of its customers and encourage them to engage with service processes. As customers can provide external resources for service innovation, especially in the context of COVID-19, the hotels should provide reward for customers who offer good advice and show cooperative behaviors. Moreover, to effectively encourage customers, hotel management should improve communication with the customers, so that hotels can more fully understand the needs of customers and build good customer partnerships.

Second, creative role identities are associated with innovation behavior, so the hotel should pay attention to the role identification of employees. According to our study, there are two ways that the hotels can improve their employees’ creative role identities. According to Farmer et al. (2003), employees who reported being creative in the past also had a stronger sense of a creative role identity, so the hotels should provide employees with ample opportunities to be creative. Further, under the background of COVID-19, the creativity of customers with epidemic prevention experience in safety and prevention and control of epidemic situation is particularly important, hotel managers should look to recruit employees who consider creativity a central part of their self-identification.

Third, creating social psychological climates is of the utmost importance to employee innovation behavior in hotels (Li and Hsu, 2016a). Our study confirms that innovation climates enhance the relationship between creative role identities and employee innovation behavior. As Shanker et al. (2017) have pointed out, managers who want to strengthen or develop a strong climate for innovation must be aware of issues that need to be taken into consideration. Therefore, hotels should take measures to improve their innovation climates. For example, hotels can improve and perfect innovation mechanisms and provide innovation reward to employees. Particularly in the Chinese cultural context, leaders have greater authority, so hotel managers should play a leading role in the process of innovation.

### Limitations and Future Research Directions

Despite its contributions, this research has several limitations. First, the sampling in our research only considered four and five-star hotels in Hangzhou city and this may weaken the generalizability of the research findings. Other cities, especially in less developed areas, and other types of hotels, such as economy hotels, should be the focus of future research. Another limitation on sampling may be the common method bias. Our core constructs were measured at the same time-wave, and future research may seek to collect data from a different time-wave to improve the measurement scales as well as the models in a hospitality context.

Second, under the attitude-intention-behavior framework, we only considered the creative role identity as an employee intention and motivation component and ignored the potential effects of other factors such as job satisfaction (Zhao et al., 2016), and tangible and intangible reward (Yoon et al., 2015). We remain unsure of whether the effect of customer cooperation would be the same if other types of intention and motivation were included. Because employees often experience different kinds of creative intention and motivation, future research should investigate different types of mediation mechanisms that exist between customer cooperation and employee innovation behavior.

Third, the match between customers and employees could vary by organizational climate. We only considered the innovation climate as an environmental factor that might influence the effectiveness of creative role identity. Organizational climates not only include the innovation climate, but also include other types of climate such as the political influence climate (Christiansen et al., 1997) and the climate for inclusion (Nishii, 2013). Future research could test different kinds of climate to verify the unique and incremental contribution of customer cooperation and creative role identity beyond these potential factors that influence employee innovation behavior. Customers in the hospitality industry display more behaviors than the cooperative. Future research could also investigate the effect of other customer-related constructs, such as customer voice and creative process engagement (Zhang and Bartol, 2010). These issues together present interesting topics for future research.

## Data Availability Statement

The original contributions presented in the study are included in the article/supplementary material, further inquiries can be directed to the corresponding author/s.

## Ethics Statement

Ethical review and approval was not required for the study on human participants in accordance with the local legislation and institutional requirements. Written informed consent for participation was not required for this study in accordance with the national legislation and the institutional requirements. Written informed consent was obtained from the individual(s) for the publication of any potentially identifiable images or data included in this article.

## Author Contributions

JZ was responsible for idea generation, data analysis, manuscript writing for theoretical part, and data collection. JY was responsible for idea generation and manuscript revision. XZ was responsible for initial method part writing. All authors contributed to the article and approved the submitted version.

## Conflict of Interest

The authors declare that the research was conducted in the absence of any commercial or financial relationships that could be construed as a potential conflict of interest.
